# Supplementary material for: Archival bone marrow smears are useful in targeted next-generation sequencing for diagnosing myeloid neoplasms
Source: PLoS One. 2021 Jul 23;16(7):e0255257. doi: 10.1371/journal.pone.0255257 (PMC8301613; doi:10.1371/journal.pone.0255257)
Supplement: S1 Table — (DOC) [file pone.0255257.s001.doc]

**S1 Table.** Paired samples of bone marrow cells and smears

| Patient | Clinical diagnosis | Smear sample | BMC sample | Elapsed years |
| --- | --- | --- | --- | --- |
| #106 | MDS | Unstained | Frozen | 4 |
| #113 | MDS | MGG-stained | Frozen | 11 |
| #181 | MDS suspected | Unstained | Fresh | Fresh |
| #184 | AA | Unstained | Fresh | Fresh |
| #220 | t-AML | Unstained | Frozen | 0.1 |

MDS, myelodysplastic syndrome; AA, aplastic anemia; t, therapy-related; AML, acute myeloid leukemia; MGG, May-Grünwald Giemsa

**Table 2.** List of patients, sample status, and pathogenic genes detected in the DNA/RNA target sequencing data of the smear samples

| Patient | | Clinical diagnosis | | staining | | Elapsed  years | | Mutation | | | | | | | |  | |
| --- | --- | --- | --- | --- | --- | --- | --- | --- | --- | --- | --- | --- | --- | --- | --- | --- | --- |
|  |  |  |  |  |  |  |  | Gene | | Nucleic acid | | Amino acid | | VAF (%) | |  | |
| #097 | AML | | no | | 0.2 | | *-* | | - | | - | | - | |  | |  |
| #112 | AML | | no | | 0.1 | | *NRAS* | | c.181C>A | | p.Gln61Lys | | 40.21 | |  | |  |
| #220 | t-AML | | no | | 0.1 | | *NRAS* | | c.182A>G | | p.Gln61Arg | | 46.84 | |  | |  |
| #226 | AML | | MGG | | 0.1 | | *CEBPA* | | c.912_913insTTG | | p.Lys304_Gln305insLeu | | 88.66 | |  | |  |
|  |  |  |  |  |  |  | *FLT3* | | c.1747_1794dup | | ITD16aa | | 14.89 | |  | |  |
| #152 | AML-MRC | | MGG | | 0.2 | | *-* | | - | | - | | - | |  | |  |
| #111 | AML | | no | | 0.2 | | *FLT3* | | c.1836_1837insdup* | | ITD17aa | | 12.53 | |  | |  |
| #161 | AML | | no | | 0.1 | | *IDH1* | | c.395G>A | | p.Arg132His | | 44.53 | |  | |  |
| #175 | AML | | no | | 0.1 | | *TP53* | | c.488A>G | | p.Tyr163Cys | | 39.92 | |  | |  |
| #176 | AML | | no | | 0.2 | | *ASXL1* | | c.1605dupT | | p.Pro536SerfsTer8 | | 21.31 | |  | |  |
|  |  |  |  |  |  |  | *FLT3* | | c.2039C>T | | p.Ala680Val | | 10.80 | |  | |  |
| #232 | AML | | no | | 0.3 | | *SRSF2* | | c.284C>A | | p.Pro95His | | 51.47 | |  | |  |
|  |  |  |  |  |  |  | *NPM1* | | c.860_863dupTCTG | | p.Trp288CysfsTer12 | | 46.70 | |  | |  |
| #256 | AML | | no | | 0.3 | | *CEBPA* | | c.917_934delGCAAC  GTGGAGACGCAGC | | p.Arg306_Gln311del | | 49.78 | |  | |  |
|  |  |  |  |  |  |  | *CEBPA* | | c.350dupG | | p.Ala118ArgfsTer52 | | 43.03 | |  | |  |
|  |  |  |  |  |  |  | *WT1* | | c.1223T>A | | p.Leu408Ter | | 83.64 | |  | |  |
|  |  |  |  |  |  |  | *GATA2* | | c.949A>G | | p.Asn317Asp | | 44.52 | |  | |  |
| #191 | AML | | no | | 0.3 | | *NRAS* | | c.34G>A | | p.Gly12Ser | | 37.97 | |  | |  |
|  |  |  |  |  |  |  | *TET2* | | c.4144delC | | p.His1382ThrfsTer66 | | 46.88 | |  | |  |
|  |  |  |  |  |  |  | *TET2* | | c.1842dupG | | p.Leu615AlafsTer23 | | 38.29 | |  | |  |
|  |  |  |  |  |  |  | *NPM1* | | c.863_864insCATG | | p.Trp288CysfsTer12 | | 44.12 | |  | |  |
|  |  |  |  |  |  |  | *PTEN* | | c.802-2A>T | | Splicing | | 5.51 | |  | |  |
| #205 | AML | | MGG | | 1 | | *SRSF2* | | c.284_307del | | p.Pro95_Arg102del | | 45.87 | |  | |  |
|  |  |  |  |  |  |  | *IDH2* | | c.419G>A | | p.Arg140Gln | | 33.72 | |  | |  |
|  |  |  |  |  |  |  | *STAG2* | | c.1810C>T | | p.Arg604Ter | | 27.27 | |  | |  |
|  |  |  |  |  |  |  | *STAG2* | | c.2534-1G>A | | Splicing | | 6.69 | |  | |  |
| #153 | AML-MRC | | MGG | | 0.1 | | *DDX41* | | c.1496dupC | | p.Ala500CysfsTer9 | | 48.83 | |  | |  |
|  |  |  |  |  |  |  | *DDX41* | | c.1574G>A | | p.Arg525His | | 12.16 | |  | |  |
|  |  |  |  |  |  |  | *SRSF2* | | c.284C>G | | p.Pro95Arg | | 11.69 | |  | |  |
| #188 | aCML | | MGG | | 1 | | *KRAS* | | c.35G>T | | p.Gly12Val | | 37.97 | |  | |  |
| #101 | MDS | | MGG | | 0.3 | | *U2AF1* | | c.101C>T | | p.Ser34Phe | | 31.77 | |  | |  |
| #113 | MDS | | MGG | | 11 | | *ATM* | | c.3078delG | | p.Trp1026CysfsTer3 | | 6.09 | |  | |  |
| #233 | MDS | | MGG | | 0.1 | | *TP53* | | c.659A>G | | p.Tyr220Cys | | 18.65 | |  | |  |
|  |  |  |  |  |  |  | *TP53* | | c.586C>T | | p.Arg196Ter | | 15.30 | |  |  |  |
| #119 | MDS | | no | | 0.4 | | *RUNX1* | | c.417C>A | | p.Asn139Lys | | 48.05 | |  | |  |
|  |  |  |  |  |  |  | *RUNX1* | | c.610C>T | | p.Arg204Ter | | 29.65 | |  | |  |
|  |  |  |  |  |  |  | *EZH2* | | c.458A>G | | p.Tyr153Cys | | 40.73 | |  | |  |
| #189 | MDS | | MGG | | 0.3 | | *TP53* | | c.817C>T | | p.Arg273Cys | | 17.34 | |  | |  |
|  |  |  |  |  |  |  | *ASXL1* | | c.2350delG | | p.Asp784MetfsTer34 | | 3.04 | |  | |  |
| #106 | MDS | | no | | 4 | | *-* | | - | | - | | - | |  | |  |

AML, acute myeloid leukemia; t, therapy-related; AML-MRC, AML with myelodysplasia-related changes; aCML, atypical chronic myeloid leukemia; MDS, myelodysplastic syndromes; MGG, May-Grünwald Giemsa; NA, not available. *: c.1836_1837insCGGC1788_1836dup

**Table 3.** List of patients, karyotypes, and fusion genes detected in the RNA target sequencing data of the smear samples

| Patient | Clinical diagnosis | Karyotype | Mapped  reads | Detected fusion gene | RPM |
| --- | --- | --- | --- | --- | --- |
| #097 | AML | 46,XY,t(8;21)(q22;q22)[17]/46,XY[3] | 33369 | *RUNX1-RUNX1T1* | 13828 |
| #112 | AML | 46,XX,inv(16)(p13.1q22)[20] | 35387 | *CBFB-MYH11* | 2560 |
| #152 | AML-MRC | 46,XX[20] | 48535 | *KMT2A-MLLT10* | 1551 |
| #176 | AML | 46,XY[20] | 44467 | - | - |
| #188 | aCML | 47,XY,+6[20] | 20447 | - | - |
| #191 | AML | 46,XX[20] | 84383 | - | - |
| #205 | AML | 46,XY[20] | 27515 | - | - |
| #220 | t-AML | 46,XX,inv(16)(p13.1q22)[20] | 48261 | *CBFB-MYH11* | 2546 |
| #226 | AML | 46,XX,i(7)(p10),-9,-9,+mar1,+mar2[20] | 31830 | - | - |
| #231 | MPAL | 46,XY,add(17)(p11.2)[12]/  46,XY,del(17)(p?)[6]/46,XY[2] | 167249 | *NUP214-ABL* | 2255 |
| #232 | AML | 46,XY,del(11)(p?)[1]/46,XY[19] | 50825 | - | - |
| #238 | AML-MRC | 46,XY,t(4;12)(q12;p13)[14]/46,XY[6] | 30749 | *ETV6-CHIC2* | 1524 |
| #240 | AML | 46,XY,t(8;21)(q22;q22.1)[3]/46,idem,-Y[14]/  46,idem,del(9)(q?)[2]/46,XY[1] | 56151 | *RUNX1-RUNX1T1* | 166556 |
| #248 | AML | 46,XX,+8[2]/46,XX[18] | 13875 | - | - |
| #256 | AML | 47,XY,+10[3]/46,XY[17] | 35635 | - | - |

AML, acute myeloid leukemia; t, therapy-related; AML-MRC, AML with myelodysplasia-related changes; aCML, atypical chronic myeloid leukemia; MGG, May-Grünwald Giemsa; MPAL, mixed phenotype acute leukemia, t-AML, therapy-related acute myeloid leukemia.

**Table S1.** Target genes for targeted sequencing

| AmpliSeq Illumina Myeloid Panel (DNA) | | |  | Custom designed panel (DNA) | | |  | AmpliSeq Illumina Myeloid Panel (RNA) | | |
| --- | --- | --- | --- | --- | --- | --- | --- | --- | --- | --- |
| Region | Genes | |  | Region | Genes | |  | Region | Genes | |
| Partial* | ABL1 | KRAS |  | Full-length | ALK | KMT2A |  | Fusion driver** | ABL1 | MET |
|  | BRAF | MPL |  |  | APC | KMT2D |  |  | ALK | MLLT10 |
|  | CBL | MYD88 |  |  | ATM | LRP1B |  |  | BCL2 | MLLT3 |
|  | CSF3R | NPM1 |  |  | ATRX | MTOR |  |  | BRAF | MYBL1 |
|  | DNMT3A | NRAS |  |  | BCORL1 | NOTCH1 |  |  | CCND1 | MYH11 |
|  | FLT3 | PTPN11 |  |  | CDKN2A | NOTCH2 |  |  | CREBBP | NTRK3 |
|  | GATA2 | SETBP1 |  |  | CUX1 | NTRK3 |  |  | EGFR | NUP214 |
|  | HRAS | SF3B1 |  |  | DDX41 | PTEN |  |  | ETV6 | PDGFRA |
|  | IDH1 | SRSF2 |  |  | ERBB2 | RAD21 |  |  | FGFR1 | PDGFRB |
|  | IDH2 | U2AF1 |  |  | ERBB3 | RET |  |  | FGFR2 | RARA |
|  | JAK2 | WT1 |  |  | FBXW7 | SMC1A |  |  | FUS | RBM15 |
|  | KIT |  |  |  | JAK3 | SMC3 |  |  | HMGA2 | RUNX1 |
| Full-length | ASXL1 | STAG2 |  |  | KDM6A | SPEN |  |  | JAK2 | TCF3 |
|  | BCOR | TET2 |  |  | KDR | TET1 |  |  | KMT2A | TFE3 |
|  | CALR | TP53 |  |  |  |  |  |  | MECOM |  |
|  | CEBPA | ZRSR2 |  |  |  |  |  |  |  |  |
|  | ETV6 | NF1 |  |  |  |  |  |  |  |  |
|  | EZH2 | PRPF8 |  |  |  |  |  |  |  |  |
|  | IKZF1 | RB1 |  |  |  |  |  |  |  |  |
|  | PHF6 | SH2B3 |  |  |  |  |  |  |  |  |
|  | RUNX1 |  |  |  |  |  |  |  |  |  |

* Target regions are designated by Illumina, Inc.

** Target regions and partner genes are designated by Illumina, Inc.
