# Supplementary figures and images for: Archival bone marrow smears are useful in targeted next-generation sequencing for diagnosing myeloid neoplasms
Source: PLoS One. 2021 Jul 23;16(7):e0255257. doi: 10.1371/journal.pone.0255257 (PMC8301613; doi:10.1371/journal.pone.0255257)

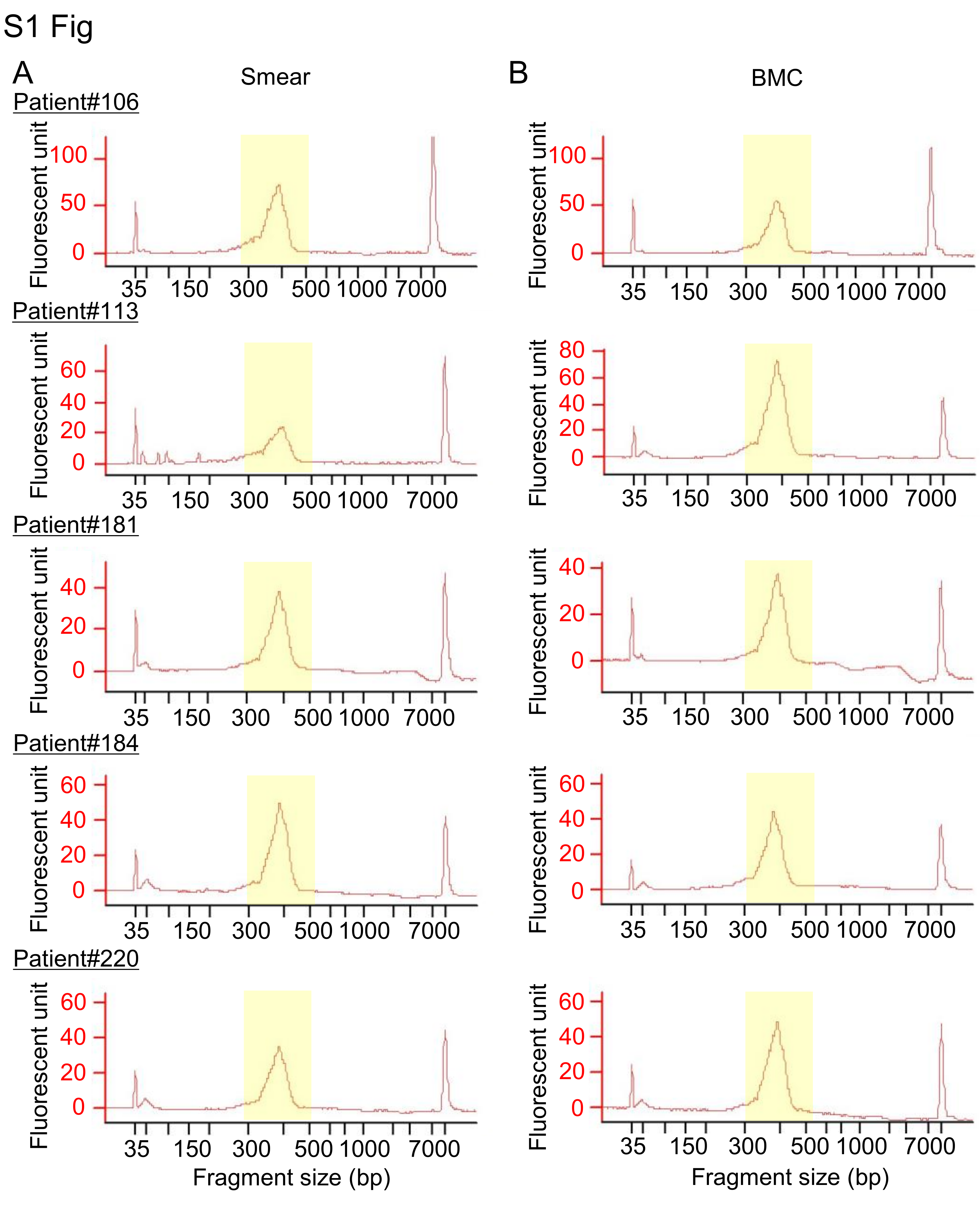

Supplement: S1 Fig — The fragment size (X axis) and fluorescent unit (Y axis) of synthesized libraries using smear-derived DNA (A) and bone marrow cell (BMC)-derived DNA (B) are shown. Yellow-highlighted regions indicate the predicted library size. (TIF) [file pone.0255257.s002.tif]

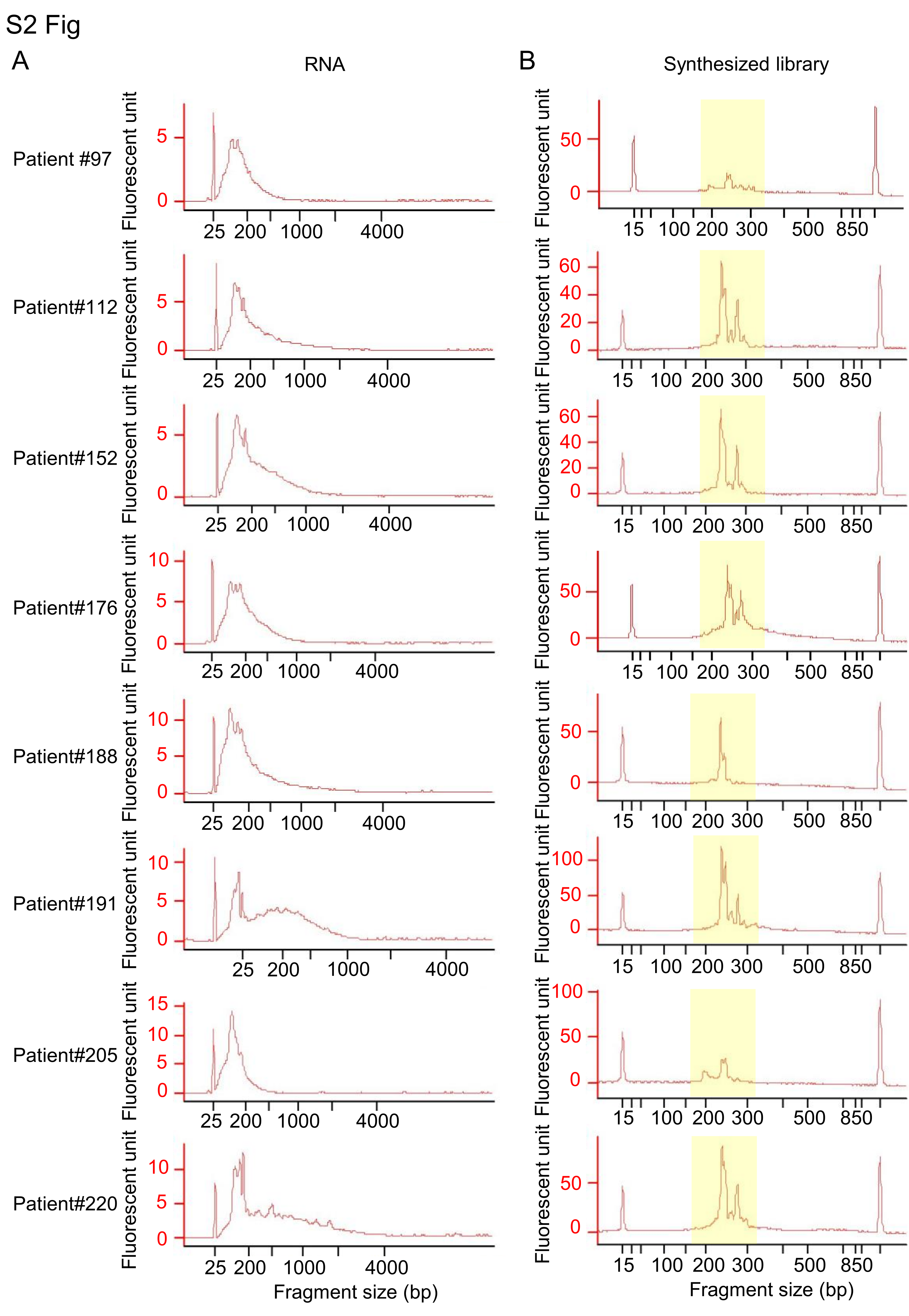

Supplement: S2 Fig — The fragment size (X axis) and fluorescent units (Y axis) of RNA (A) and the synthesized libraries (B) are shown. Yellow-highlighted regions indicate the predicted library size. (TIF) [file pone.0255257.s003.tif]

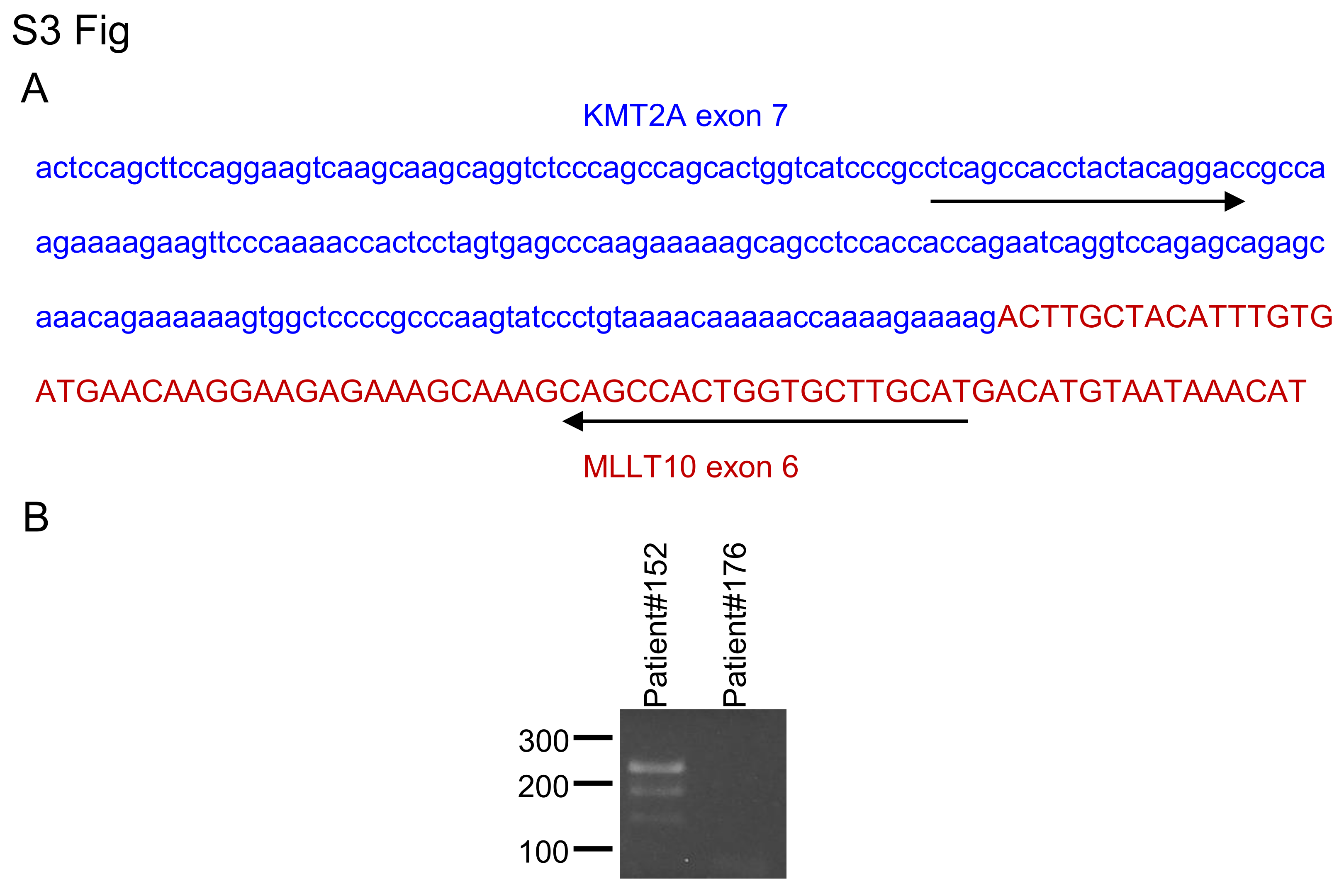

Supplement: S3 Fig — (A) The fusion sequence detected by targeted RNA sequencing is shown. Arrows indicate the primers for amplifying the target region. (B) A fusion gene confirmed by RT-PCR is shown. The following parameters were used with the PrimeSTAR GXL DNA Polymerase (TAKARA): 98°C for 3 min, followed by 35 cycles at 98°C for 10 s, 70°C for 15 s, and 68°C for 30 s. The sample from Patient#176 was used as a negative control. (TIF) [file pone.0255257.s004.tif]

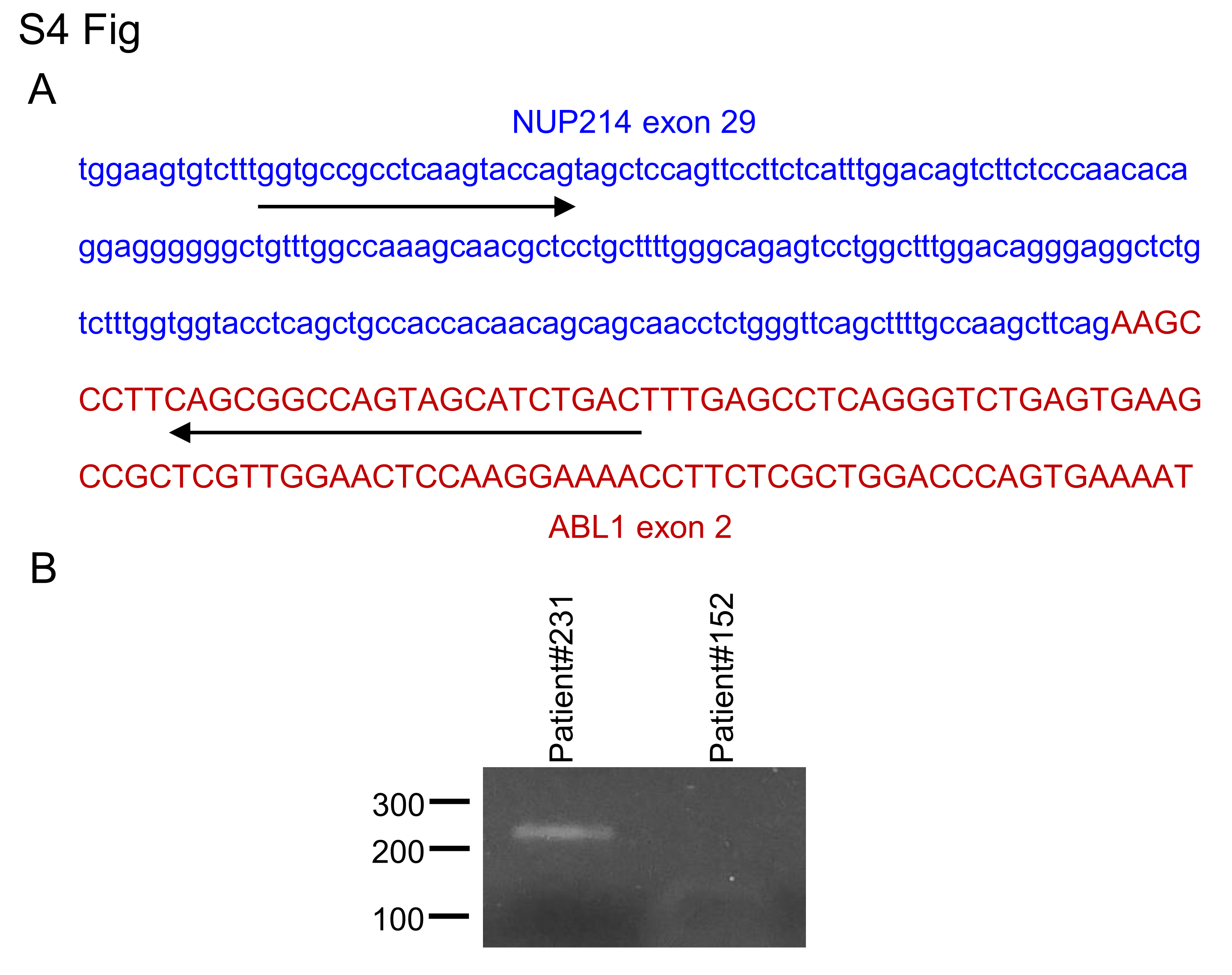

Supplement: S4 Fig — (A) The fusion sequence detected by targeted RNA sequencing is shown. Arrows indicate the primers used to amplify the target region. (B) A fusion gene confirmed by RT-PCR is shown. The following parameters were used with the PrimeSTAR GXL DNA Polymerase (TAKARA): 98°C for 3 min, followed by 35 cycles at 98°C for 10 s, 75°C for 15 s, and 68°C for 30 s. The sample from Patient#176 was used as a negative control. (TIF) [file pone.0255257.s005.tif]

Raw image of S3B Fig

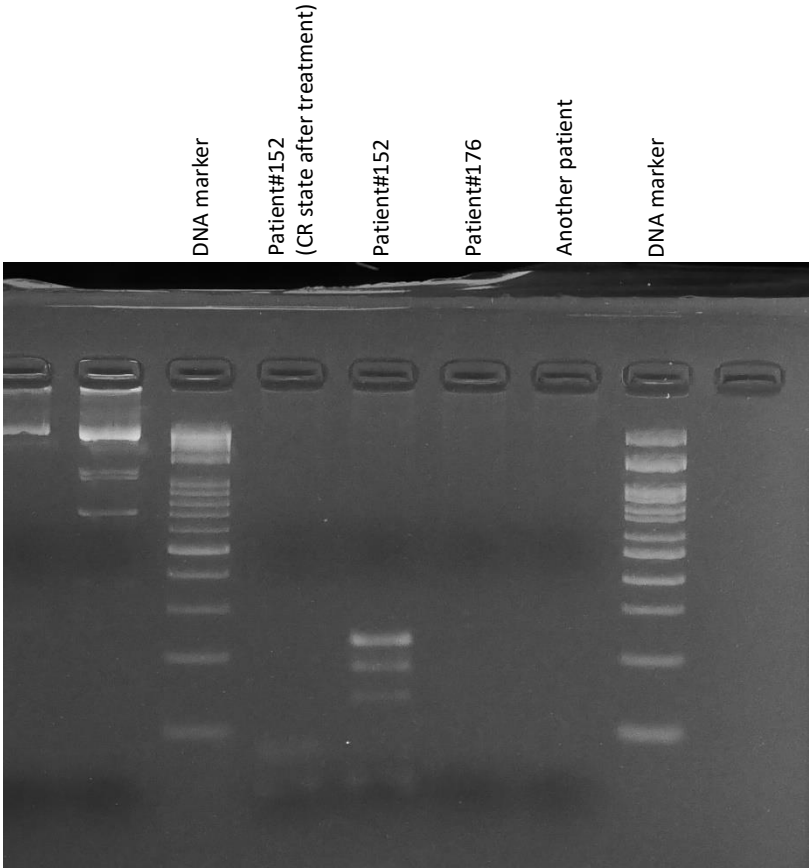

Used in S3 Fig

Raw image of S4B Fig

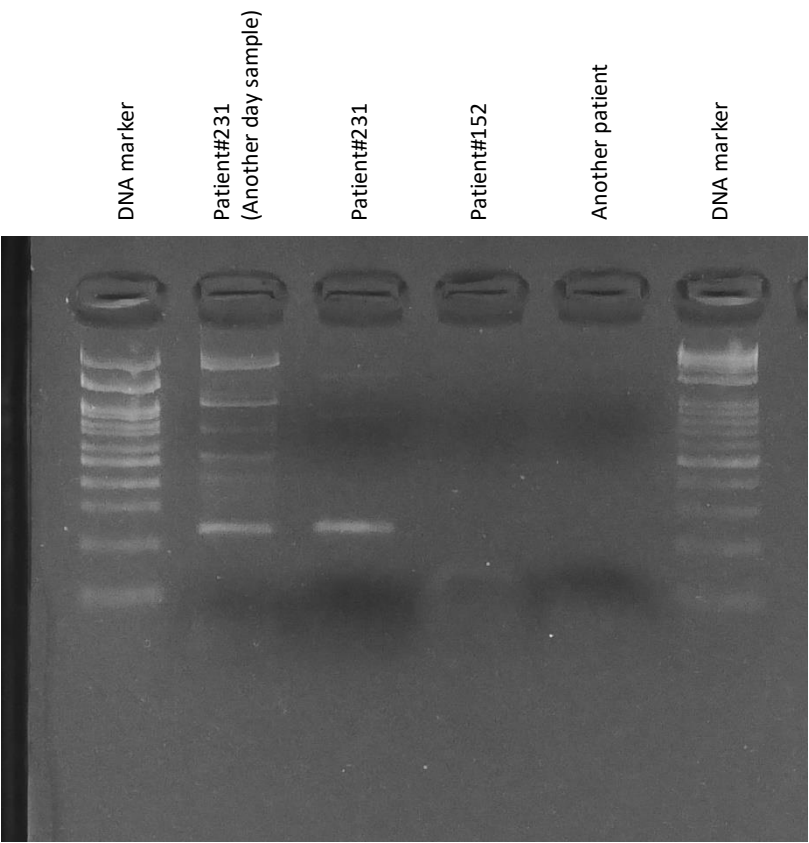

Used in S4 Fig

Supplement: S1 Raw images — (PDF) [file pone.0255257.s006.pdf]
