# Supplementary material for: Archival bone marrow smears are useful in targeted next-generation sequencing for diagnosing myeloid neoplasms
Source: PLoS One. 2021 Jul 23;16(7):e0255257. doi: 10.1371/journal.pone.0255257 (PMC8301613; doi:10.1371/journal.pone.0255257)
Supplement: Attachment — Submitted filename: Response to Reviewers.docx [file pone.0255257.s007.docx]

Reviewer #1: Sadato et al reported very interesting result evaluating bone marrow slide as a potential source of DNA and RNA extraction for next-generation sequencing. This data is worthy of being shared with the community, but I am not sure it fits into the scope of PLOS ONE for publication. The novelty is relatively low-intermediate. The followings are my comment on the paper.

Thank you for your insightful comments, which have helped us significantly improve the paper. We added some new data and figure to address your questions below.
One of the critiques is consideration of time from marrow slide smear to the procurement for DNA/RNA extraction. As we are aware, DNA/RNA is degraded quickly over time. The authors presented elapsed time in Tables 1 & 2. But they have not analyzed the impact of elapsed time on the quality of DNA/RNA or the result of variant calling in DNAseq or fusion gene detection in RNAseq. This analysis is to be performed. I am not convinced that they have taken this part into consideration during their analysis of the data.

Also, the result comparing unstained smear slide vs. MGG stained slide is lacking. This information was presented in part, but I do not think it was analyzed systematically and compared between the two sources.

We appreciate your observations and agree. We assessed the quality of all the DNA and RNA samples used in this study and analyzed the effect of the duration of preservation and staining. We added the findings in a new figure and described them in revised manuscript as follows.

・About the DNA

As a result of measuring and comparing the dsDNA/Total DNA ratio, we found that the quality of the DNA was not significantly affected by the length of preservation or by staining. However, for variant calling, samples with longer preservation times tended to have increasing quantities of noise that were able to be excluded by filtering. These results suggested that DNA could be extracted from various smears and that variant analysis could be done as long as allowances are made for the increased noise. We newly created Fig. 8 to show these results and added a discussion of this to Results and Discussion section (page 13, line 218 to page 14, line 229), and Conclusion section (page 20, line 296).

・About the RNA

We performed a new assessment of RNA quality using the RNA integrity number (RIN) with TapeStation (Agilent Technologies). The results are shown in Fig. 9A (newly added). The RIN values were generally very low, suggesting that RNA rapidly degraded as soon as the preparation of the smears but that some level of RNA quality, however low, could be maintained during storage. And as a result, the targeted RNA sequencing was successful. We added a discussion about the methods we used to the Materials and Methods section (page 5, line 78-80) and included the results in the Results and Discussion section (page 17, line 267-269) of the revised manuscript.
Also, given that slide storage is critical for the prevention of decay and degradation of genetic material, it would be critical to describe your method of slide storage in the paper.

As pointed out, the conditions of slide storage are important information. The smear slides used in this study were stored in a clinical laboratory (at room temperature in a dark place) with no other special care. We added this information to Materials and Methods (page 5, line 72), Results and Discussion (page 13, line 207-208).
The quality of Figures was very low.

We apologize for the quality of the figures. We replaced them all with high resolution images.

Reviewer #2: Dr. Daichi Sadato and co-workers describe the use of bone marrow smears as a source of DNA and RNA for next generation sequencing analysis. Despite the inferior quality of the smear-derived material compared to nucleic acid obtained from paired fresh samples, the genetic analyses could be performed with success.
Interestingly, also extracted RNA provided useful information on known and unknown translocation. This is a nice piece of information, especially for retrospective studies or for patients with low material availability at diagnosis.

Thank you for your careful reading and helpful comments on our study. We analyzed and re-checked the data to confirm our results.

Major point:
-The HaplotypeCaller tool (for germline or large clone variant) found low percentage of smear and BMC-unique variants testifying a good correlation between variants found in fresh and smear derived material. Conversely, the Mutect2 tool (for somatic mutation) found much more variants (two-thirds of total) as smear-unique variants. N
DNA single nucleotide variants (SNV) are per se neutral and stochastic events, therefore author should try to better explain the difference between the results of smear-unique mutations between the two bioinformatic tools.

We appreciate the reviewer's observation on this point. The difference in the quantity of smear-unique mutations is due to different levels of sensitivity of the assessment tools to low VAF variants. In principle, Mutect2 can detect more low-VAF variants and thus more smear-sample-derived noise than HaplotypeCaller. We added this observation to Results and Discussion (page 13, line 208-213).
